# Keeping Surgeons “In the Fight”: Surgical Operational Stress Control, A Novel Adaptation From the US Marine Corps

**DOI:** 10.1097/SLA.0000000000006989

**Published:** 2025-11-25

**Authors:** Maro M. Doce, Omin Kwon, Elizabeth A. Pomfret, Sunil K. Geevarghese

**Affiliations:** 1 Vanderbilt University School of Medicine, Nashville, TN; 2 3rd Infantry Division, Fort Stewart/Hunter Army Airfield, Fort Stewart, GA; 3 Department of Surgery, University of Colorado, Aurora, CO; 4 Department of Surgery, Vanderbilt University Medical Center, Nashville, TN

**Keywords:** moral injury, surgeons, surgery, occupational stress, burnout, combat and operational stress control, surgical operational stress control, surgical education, psychological resilience, peer support, second victim syndrome

While there are inherent differences in stressors encountered by operational military units and surgeons, several parallels run between the professions. Both must perform effectively in high-stress environments where the safety and well-being of others are at stake. Consequently, operational military units and surgeons are both at risk for incurring different stress-related sequelae, including moral injury, burnout, and mental health disorders.^1–3^

Moral injury originally describes a form of ethical distress that military personnel may experience on the battlefield. Examples include carrying out orders at odds with personal values or harm incurred to bystanders or comrades due to one’s actions.^4^ The term has been broadened to other contexts where there is a discrepancy between one’s values and the outcome. In surgery, moral injury may be experienced when patients suffer complications and death.^1^

Moral injury in the military setting is associated with post-traumatic stress disorder, depression, and suicidality.^4,5^ For surgeons, moral injury is associated with loss of confidence, increased medical errors, and second victim syndrome.^1,6^ In surgical and military settings, it is crucial to identify and manage occupational stressors to mitigate these sequelae. For this purpose, the US military currently utilizes Combat and Operational Stress Control (COSC), a doctrinal system designed to manage traumatic events and stressors in an operational setting. Its principles can be adapted to surgery to mitigate occupational stress and moral injury among surgeons.

## HISTORY OF COMBAT OPERATIONAL STRESS CONTROL (COSC)

While the psychological effects of war have been described throughout history, they were not considered from a clinical perspective until the 18th century.^7^ As military clinicians sought to understand this phenomenon during World War I, they coined the term “shell shock.”^7^ Allied forces observed that individuals treated for “shell shock” far from the front lines rarely recovered enough to return to combat. When immediately treated in close proximity to the fight, individuals more consistently recovered functional capacity, establishing a key tenet still utilized in modern US military COSC today.^7^

## COMBAT OPERATIONAL STRESS AND CURRENT APPLICATION IN THE US MILITARY

In COSC, a “stress reaction” is defined as a temporary state of distress and functional impairment occurring as a normal response to adverse events. Left unaddressed, a temporary “stress reaction” may worsen into chronic sequelae such as a mental health disorder or moral injury.^2,4^ However, with a short course of appropriate intervention, most individuals experiencing a “stress reaction” quickly return to normal functioning.^2,7^

The US military currently employs operational stress control units, whose purpose is to maintain the well-being and function of the units they serve.^7^ COSC units are comprised of mental health, medical, and chaplain assets that interface with units and their leaders.^2^ The US Marine Corps (USMC) COSC Manual defines the 5 core functions for military leaders to maintain unit readiness: strengthen, mitigate, identify, treat, and reintegrate.^2^

At baseline, stress control units assist with the Strengthen, Mitigate, and Identify functions, assessing background factors affecting operational units, such as sleep, high operational tempo, and family stressors, to enact preventative and interventional measures.^2^ Military units may also face acute events significantly impacting morale, such as unit casualties or deaths. In these situations, COSC elements assist with the Treat and Reintegrate functions, where they render psychological first aid, assist in restoring normal unit functioning, and triage severely affected individuals requiring formal mental health care.^2^

**FIGURE 1:**
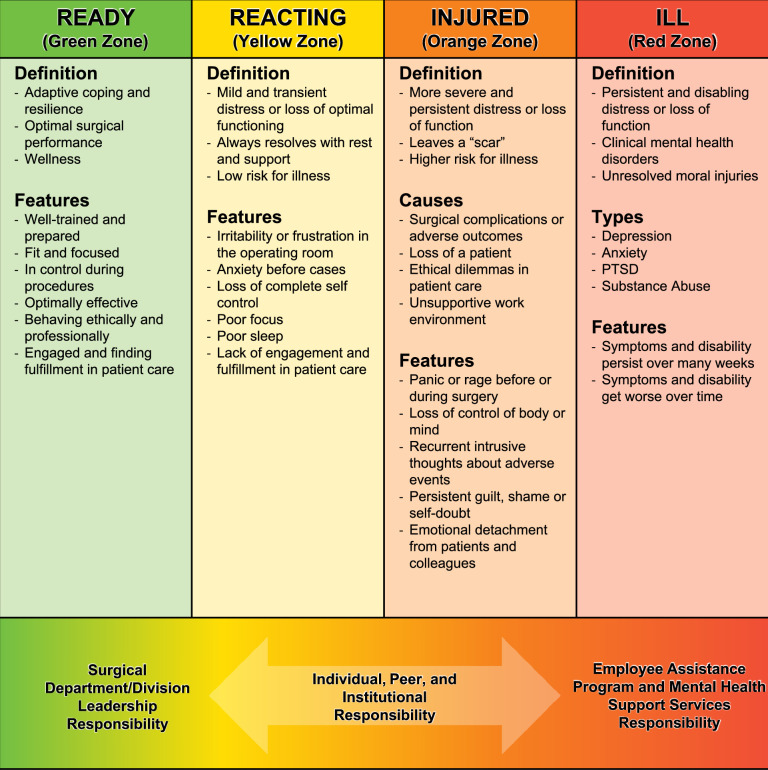
Surgical adaptation of the “Stress Continuum Model” from Marine Corps Tactical Publication (MCTP) 3-30E: Combat and Stress Control. The framework depicts the progression of stress responses among surgeons, ranging from adaptive coping (“Ready”) to severe distress and burnout (“Ill”).

In addition to these services, COSC also encompasses education for individuals and leadership. To help regular unit members recognize differing levels of distress, the USMC COSC manual outlines the “stress continuum model” (Fig. 1). In this model, “Green” describes the “ready zone,” indicating optimal functioning. “Yellow” describes the “reacting zone,” where the individual exhibits milder “stress reactions” temporarily affecting functional capacity. “Orange” describes the “injured zone,” where individuals experience stronger or prolonged “stress reactions” at risk of becoming more permanent. Lastly, “Red” describes the “ill zone,” where individuals exhibit more severe or persistent symptoms that may meet criteria for a mental health condition.^2^

## SURGICAL OPERATIONAL STRESS CONTROL (SOSC): TRANSLATING MILITARY RESILIENCE TO SURGICAL SYSTEMS

Although the specific stressors may differ, surgeons must maintain emotional control and focus under intense workloads to perform effectively in the operating room. They inevitably face challenging circumstances, including complications, poor outcomes, and patient death.^7^ Many hospitals employ wellness efforts, but they often address factors occurring outside the operating room. Peer support systems are widespread in the US now, and among them are units based in hospitals, training programs, and societies. The Vanderbilt Resilience Rapid Response Team is an example of a training program-based peer support system which started in 2018.^8^ This approach was the inspiration to create the American Society of Transplant Surgeons Peer Support System in 2020 becoming the first society-based peer support system.^1^ However, an unmet need in all systems is a standardized approach to the identification of resilience breaches in its varied forms. We believe the COSC’s battle-tested approach can be effectively and easily translated to the surgical environment of training and practice. We have termed it Surgical Operational Stress Control (SOSC).

SOSC translates the five core functions outlined in the USMC COSC Manual—Strengthen, Mitigate, Identify, Treat, and Reintegrate—to support the well-being and performance of surgeons in a highly demanding profession.

### Strengthen

This function fosters resilience and trust before surgeons encounter high-stakes clinical situations to proactively prepare individuals for the demands of surgery. Key elements include:

- Foster vertical and lateral connections through mentorship programs and peer support networks. Implementing programs that encourage open dialogue about personal and career adversities normalizes challenges inherent to surgical practice and promotes resilience through community cohesion.

### Mitigate

The second function focuses on recognizing and reducing the impact of routine stressors in surgical practice, aiming to maintain surgeons within the “green” and “yellow” stress continuum zones, and prevent escalation into the “orange” or “red” zones.

- Promote shared decision-making, especially for early career surgeons who have not yet developed an established track record to build “protective equity.”^3^ Shared responsibility enhances support, reduces cognitive burden, and fosters an environment of trust. For example, some transplantation centers utilize a joint decision model to create this shared responsibility and enable protection for junior faculty.^9^

### Identify

The third function focuses on detecting early “yellow” or “orange” zone signs of a “stress reaction” before they escalate into chronic dysfunction within the “red zone.”

- Provide structured training for surgeons to recognize a spectrum of reactions across the stress continuum (Fig. 1). This type of training increases recognition of common presentations of physician distress that may not always be openly discussed.
- Normalize conversations about stress. Open discussion about career and personal adversities not only foster cohesion and destigmatization but also enable others to recognize “stress reactions.”

### Treat

The fourth function addresses situations where a transient “stress reaction” progresses into a persistent “red zone” condition that chronically affects a surgeon’s well-being and performance.

- Establish a tiered support system that includes both peer and leadership engagement as well as pathways for accessing professional resources. Timely intervention reduces stigma and promotes recovery.
- Promote narrative reflection and finding meaning in difficult experiences by encouraging candid discussions with trusted mentors and peers. Reflective dialogue helps individuals process moral injury, restores purpose, and rebuilds resilience following distressing experiences.

### Reintegrate

The final function supports a surgeon’s return to full clinical responsibilities following a period of sustained “orange zone” or “red zone” stress. In surgical culture—where stepping back is often misinterpreted as weakness—reintegration must be intentional, structured, and stigma-free.^9^

- Allow space for recovery by developing individualized reintegration plans that gradually transition affected personnel back to full clinical duties, with consideration for pacing and readiness.
- Assign experienced mentors to guide team members through reintegration. Senior support validates the experience, provides reassurance, and normalizes recovery as a routine part of a long surgical career.
- Reframe recovery as a sign of professional resilience and adaptive growth—not as a personal or professional failure.

## APPLICATION TO SURGICAL EDUCATION

The 5 core functions of Surgical Operational Stress Control (SOSC) offer a structured framework through which the surgical community can adapt proven military principles to mitigate the unique stressors of surgical practice. In a field defined by life-and-death decision-making, repeated exposure to adverse outcomes, and unrelenting expectations, even the most resilient clinicians are vulnerable to emotional fatigue and burnout. SOSC normalizes these pressures by fostering a culture of transparency, an environment of trust, and peer support. It strengthens team cohesion and empowers early recognition, treatment, and reintegration to ensure that no surgeon faces these challenges alone. As Combat Operational Stress Control enhances the longevity of military units in battle, an integrated SOSC model has the potential to sustain surgical teams through the rigors of modern practice and keep surgeons “in the fight” where they are needed most: the operating room.
